# Supplementary material for: Multipole engineering by displacement resonance: a new degree of freedom of Mie resonance
Source: Nat Commun. 2023 Nov 8;14:7213. doi: 10.1038/s41467-023-43063-y (PMC10632421; doi:10.1038/s41467-023-43063-y)
Supplement: Supplementary file 1 — Supplementary_Information [file 41467_2023_43063_MOESM1_ESM.pdf]

Supplementary Information for

Multipole engineering by displacement resonance: a new degree of freedom of Mie resonance

Yu-Lung Tang, Te-Hsin Yen, Kentaro Nishida, Chien-Hsuan Li, Yu-Chieh Chen, Tianyue Zhang, Chi-Kang Pai, Kuo-Ping Chen, Xiangping Li*, Junichi Takahara* and Shi-Wei Chu*

Correspondence to: xiangpingli@jnu.edu.cn, takahara@ap.eng.osaka-u.ac.jp, swchu@phys.ntu.edu.tw

This PDF file includes:

Supplementary Notes 1 to 2
Supplementary Fig. 1 to 13
Supplementary References

Other Supplementary Materials for this manuscript include the following:

Supplementary Videos 1 to 2

Supplementary Notes 1: Calculation of non-paraxial excitation beam

In our simulation, we used a non-paraxial Gaussian beam produced by the built-in COMSOL function of the angular spectrum method. In the angular spectrum method, the electric field ***E(r)*** is calculated via the Helmholtz equation[1].

$$\left(\nabla^2 + k^2\right)\boldsymbol{E}(\boldsymbol{r}) = 0 \tag{S1}$$

where the electric field ***E(r)*** separated into transverse E_t and longitudinal E_z components by using unit vectors ***n(r)*** and ***z(r)***,

$$\boldsymbol{E}(\boldsymbol{r}) = \boldsymbol{n}(\boldsymbol{r})E_t + \boldsymbol{z}(\boldsymbol{r})E_z \tag{S2}$$

Here, complete solutions of E_t and E_z by expanding as a power series,

$$E_t = \psi e^{ikz} = (\sum_{n=0}^{\infty} f^{2n}\psi^{(2n)})e^{ikz} \tag{S3}$$

$$E_z = \phi e^{ikz} = (\sum_{n=0}^{\infty} f^{2n+1}\phi^{(2n+1)})e^{ikz} \tag{S4}$$

where, f is perturbation parameter which defined as:

$$f = w_0/l \tag{S5}$$

where, w_0 is the beam width and l is the diffraction length. Each component of $\psi^{(n)}$ and $\varphi^{(n)}$ in Eq.(S3) and Eq.(S4) are obtained by solving the following differential equations,

$$(2i\frac{\partial}{\partial\zeta} + \frac{\partial^2}{\partial\xi^2} + \frac{\partial^2}{\partial\eta^2})\psi^{(0)} = 0 \tag{S6}$$

$$(2i\frac{\partial}{\partial\zeta} + \frac{\partial^2}{\partial\xi^2} + \frac{\partial^2}{\partial\eta^2})\psi^{(2n)} = -\frac{\partial^2\psi^{(2n-2)}}{\partial\zeta^2} \tag{S7}$$

$$\phi^{(0)} = i\frac{\partial\psi^{(0)}}{\partial\xi} \tag{S8}$$

$$\phi^{(2n+1)} = i\frac{\partial\psi^{(2n)}}{\partial\xi} + i\frac{\partial\phi^{(2n-1)}}{\partial\xi} \tag{S9}$$

where,

$$\zeta = z/l \tag{S10}$$

$$\xi = y/w_0 \tag{S11}$$

$$\eta = z/w_0 \tag{S12}$$

We applied the following boundary condition E_b at z = 0, which corresponds to the paraxial approximation of the Gaussian beam at the focal plane, to obtain the solution of Eq. S4 and S5,

$$\psi^{(2n)}(x,y,0) = \begin{cases} E_b(x,y), \text{if } n = 0 \\ 0, \text{if } n \geq 1 \end{cases} \tag{S13}$$

$$E_b(x,y) = E_0\text{exp}[-\frac{x^2+y^2}{w_0^2} - ik\frac{x^2+y^2}{2R}] \tag{S14}$$

where R is the radius of curvature at z = 0. Then, the total electric field ***E(r)*** is obtained from Eq. S2, S4 and S5, by solving the equations numerically with the boundary condition, while taking into account the relative placement of sample and excitation within the calculation domain.

Supplementary Notes 2: Calculations of scattering cross-section and multipole analysis

We calculated the scattering cross-section by first integrating the time averaged energy flow through the collection surface defined by an NA of objective lens. The integrated result is then divided by the incoming Poynting vector to obtain a scattering cross-section[2]. Note that this definition of scattering cross-section is equivalent to the fraction of energy scattered. Therefore, it is also feasible to calculate the scattering efficiency by multiplying the geometric cross-sectional area projected onto the focal plane.

In order to performed multipole decomposition analysis (MDA), we first calculated the electric field distribution of nanostructure in the FEM numerical simulation software COMSOL Multiphysics (COMSOL Inc.), and obtained the electric current distribution by using this equation:

$$\boldsymbol{J}_{\boldsymbol{\omega}}(\boldsymbol{r}) = i\omega\varepsilon_0(\varepsilon_r - 1)\boldsymbol{E}_{\boldsymbol{\omega}}(\boldsymbol{r}) \tag{S15}$$

Then, by the approach of Ref [3], multipolar moments in the Cartesian coordinate are obtained by integration of current density in the Fourier space. The exact expression of these integration are as follows:

$$p_\alpha = -\frac{1}{i\omega}\left\{\int d^3\mathbf{r}\; J_\alpha^\omega j_0(kr) + \frac{k^2}{2}\int d^3\mathbf{r}\left[3(\mathbf{r}\ \cdot\mathbf{J}_\omega)r_\alpha - r^2 J_\alpha^\omega\right]\frac{j_2(kr)}{(kr)^2}\right\} \tag{S16}$$

$$m_\alpha = \frac{3}{2}\int d^3\mathbf{r}(\mathbf{r}\times\mathbf{J}_\omega)_\alpha\frac{j_1(kr)}{kr} \tag{S17}$$

$$\begin{aligned} Q_{\alpha\beta}^e = -\frac{3}{i\omega}\Bigg\{&\int d^3\mathbf{r}\left[3(r_\beta J_\alpha^\omega + r_\alpha J_\beta^\omega) - 2(\mathbf{r}\cdot\mathbf{J}_\omega)\delta_{\alpha\beta}\right]\frac{j_1(kr)}{kr} \\ &+2k^2\int\left[5r_\alpha r_\beta(\mathbf{r}\cdot\mathbf{J}_\omega) - (r_\alpha J_\beta + r_\beta J_\alpha)r^2 - r^2(\mathbf{r}\cdot\mathbf{J}_\omega)\delta_{\alpha\beta}\right]\frac{j_3(kr)}{(kr)^3}\Bigg\} \end{aligned} \tag{S18}$$

$$Q_{\alpha\beta}^m = 15\int d^3\mathbf{r}\left\{r_\alpha(r\times\mathbf{J}_\omega)_\beta + r_\beta(r\times\mathbf{J}_\omega)_\alpha\right\}\frac{j_2(kr)}{(kr)^2} \tag{S19}$$

where j_l is the l th order spherical Bessel function of the first kind.

Supplementary Fig 1 - 13

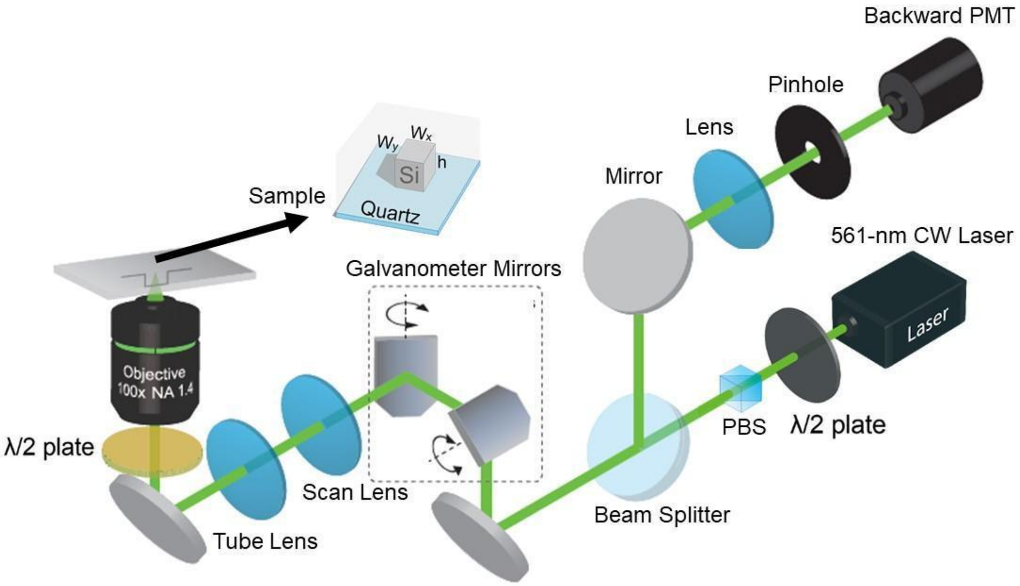

Supplementary Figure 1: Schematic of laser scanning microscope setup. The scanning system of confocal microscopy. The green light indicates the laser and backward scattering signals. The sample is crystalline Si nanocuboids on a quartz substrate immersed in oil with the same width and length ($w_x = w_y = w$), and the height (h) is fixed at 150 nm.

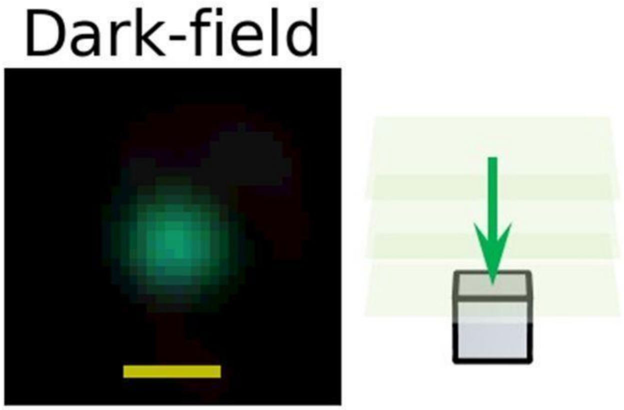

Supplementary Figure 2: Dark-field image of *w*=80 nm Si nanocuboid. The illumination source is a halogen lamp with a 550 nm band-pass filter (TBP01-550/15-25x36, Semrock). A solid circle image is observed, matching well to the conventional convolution theory based on plane wave incidence. The image forms a sharp contrast to the two-lobe image in Fig. 1c.

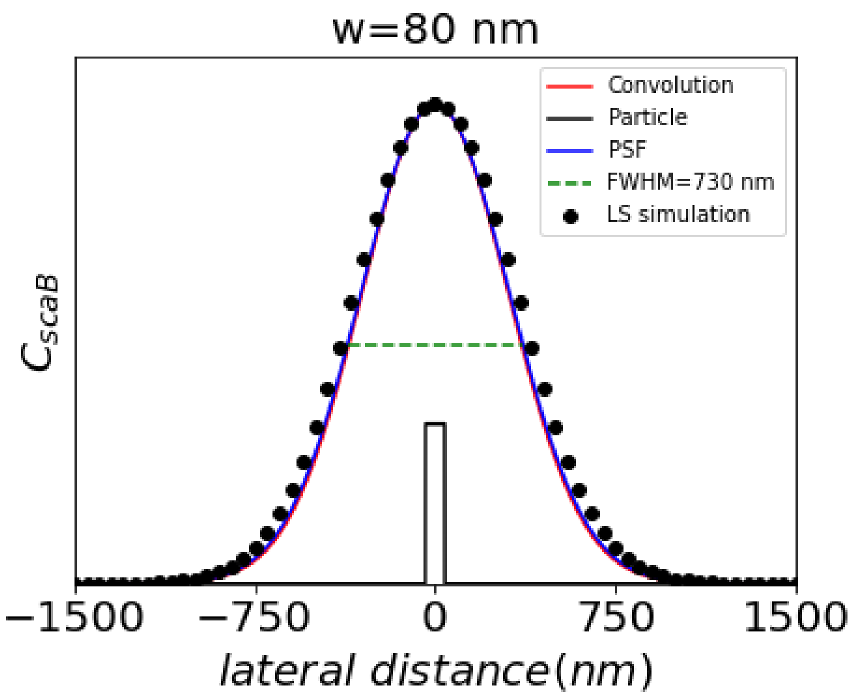

Supplementary Figure 3: LSM simulation for the loosely focused condition. Simulated LSM line profile along the x direction for the w=80 nm Si nanocuboid, whose size is schematically shown by the black line. We considered a loosely focused condition with NA = 0.4 at 561 nm. The blue line indicates the intensity profile of the excitation field (point spread function). The black dots indicate the simulated backward scattering cross-sections, matching well to the convolution results shown by the red line. Once again, this is in sharp contrast to Fig. 1c, where a two-lobe image is observed on the same particle when *w/FWHM* and *d/FWHM* are both close to unity.

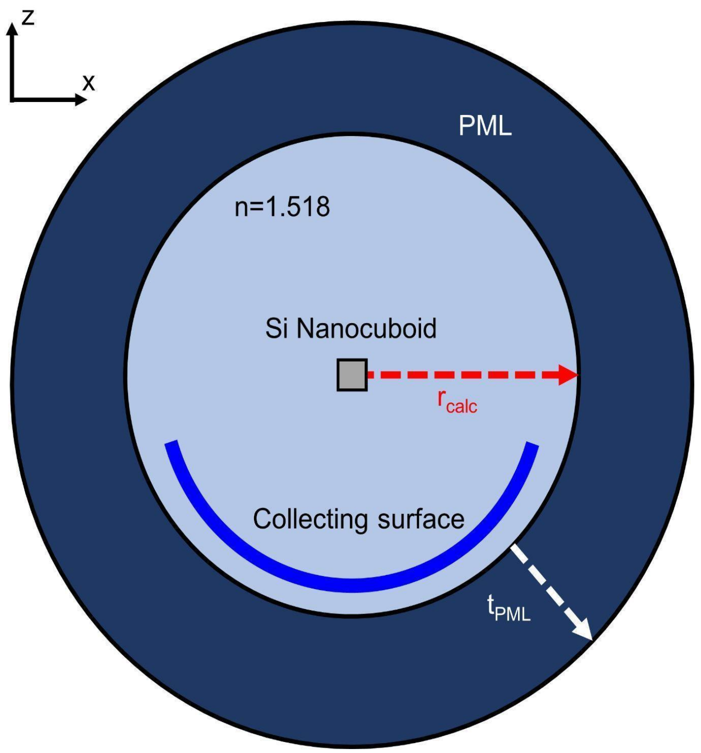

Supplementary Figure 4: Cross-section view of the simulation environment. The central gray square indicates the silicon nanocuboid placed at the center of the calculation domain. The real and imaginary refractive indices of the silicon nanocuboid were set to 3.9786 and 0.02302, which were the experimentally measured values at the excitation wavelength of 561 nm by using an ellipsometer in our previous research[4]. The radius of the calculation domain (r_{calc}) is 1200 nm. The calculation domain is surrounded by a perfect matching layer (PML) with a thickness (t_{PML}) of 600 nm. The blue arc indicates the collecting surface, which reflects the NA of the objective lens (=1.40), for scattering signal collection in the backward direction. The medium has a refractive index of n=1.518 which corresponds to the refractive index of immersion oil.

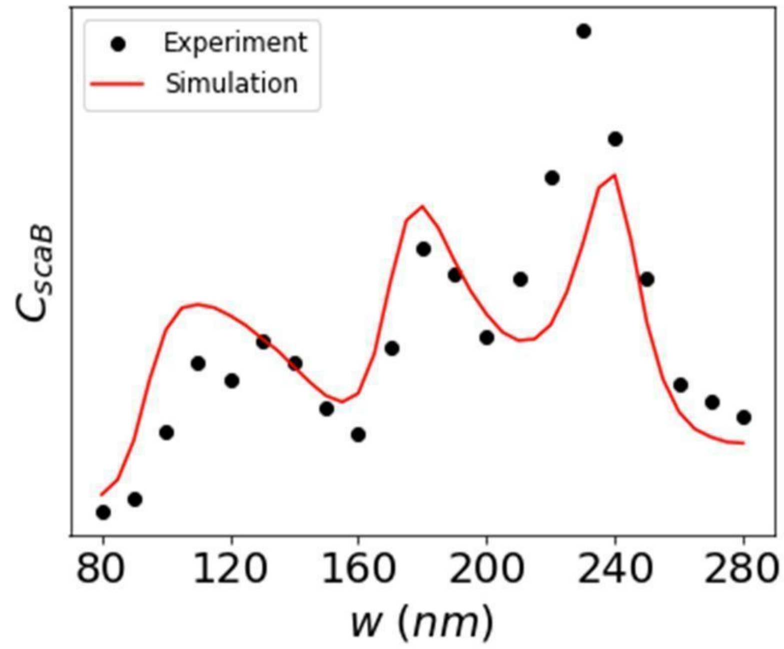

Supplementary Figure 5: Size-dependent backward scattering cross-sections at central excitation. The black dots indicate the measured backward scattering intensities, derived from the central pixel of corresponding LSM images in Fig. 2a. The red line indicates the simulated scattering cross-sections at central excitation (*d/FWHM* = 0).

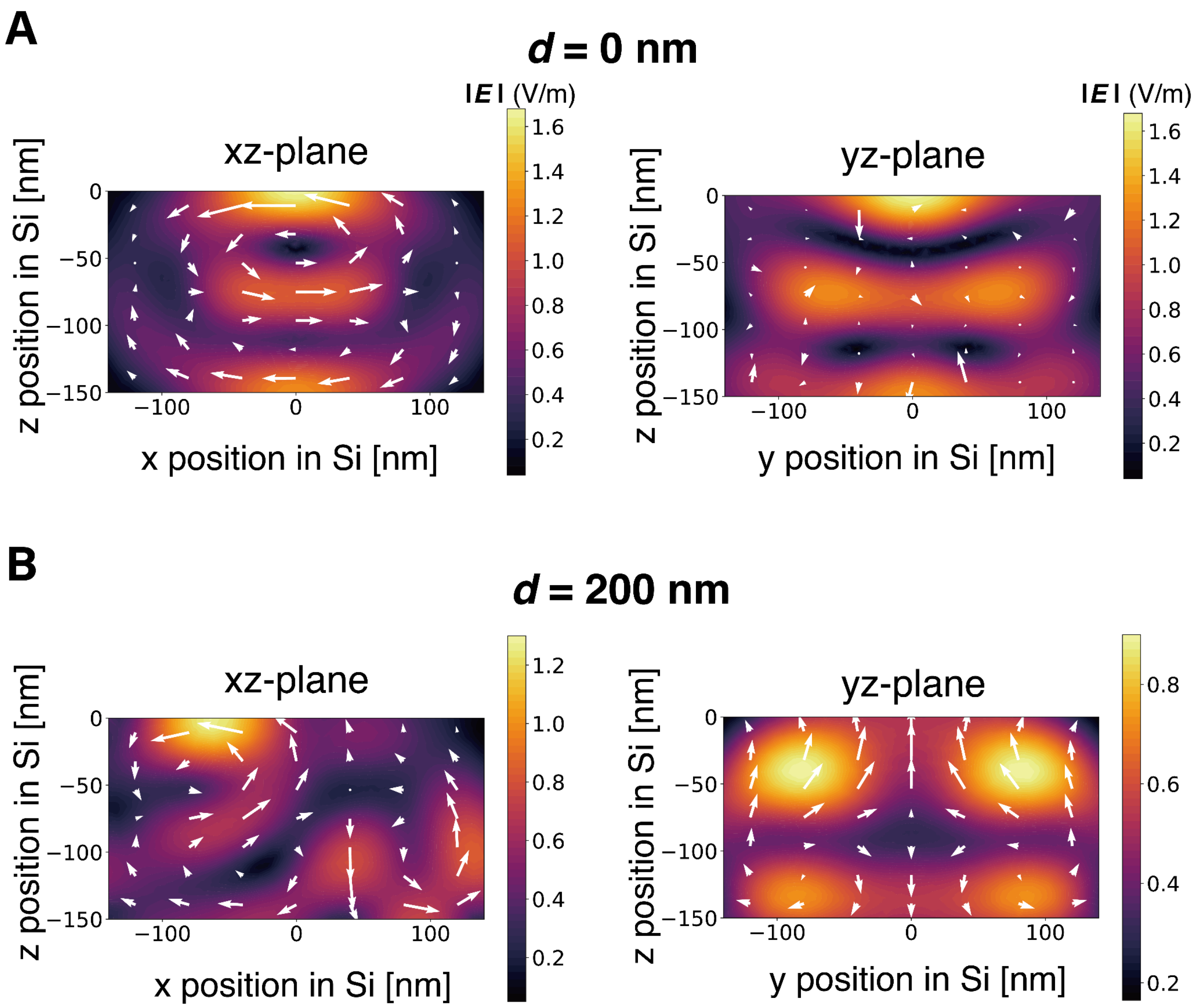

Supplementary Figure 6: Calculations of the electric field distribution inside a w=280 nm silicon nanocuboid. (A) Calculation results on non-displacement excitation (d = 0 nm). The position of the focus spot center is x = 0 nm, y = 0 nm and z= -75 nm. The left figure is xz slice of electric field distribution, circulating current and electric field show magnetic dipole resonance. The right figure is yz slice of electric field distribution, two hotspots concentrated at center show electric dipole resonance. (B) Calculation results with displacement excitation (d = 200nm). The position of the focus spot center is x = -200 nm, y = 0 nm and z= -75 nm. The xz slice show that original circulating current is disrupted due to asymmetry after beam displacement, which causes disappearance of magnetic dipole mode. The yz slice show that four hotspots at four corners appear, which correspond to electric quadrupole mode.

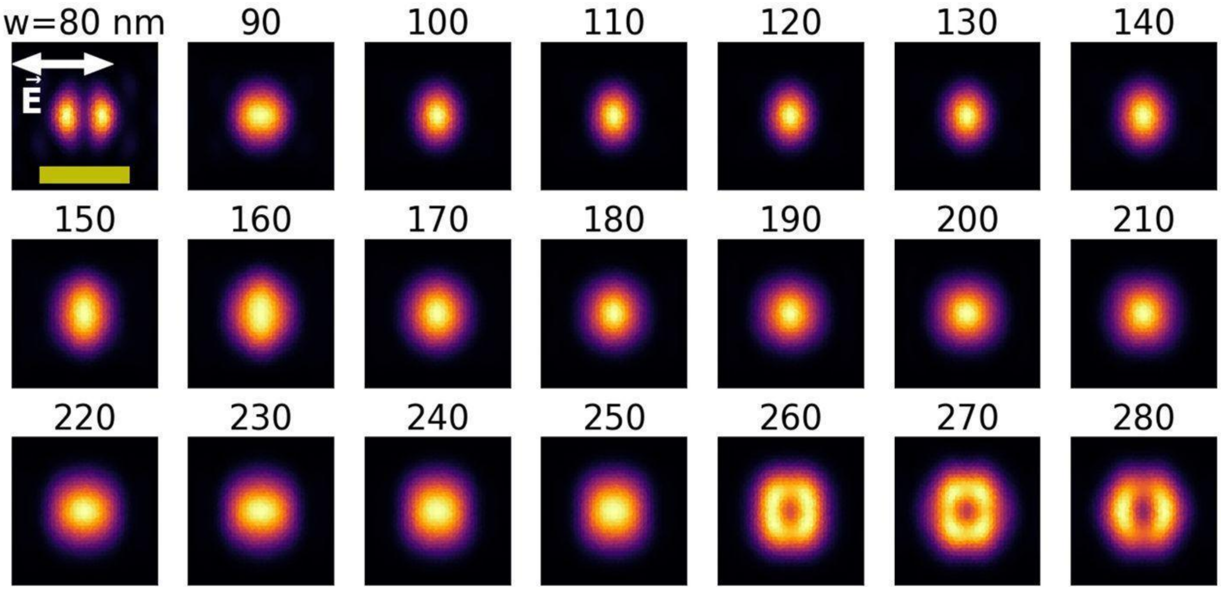

Supplementary Figure 7: LSM simulation of the whole array at horizontal polarization, corresponding to experimental results in Fig. 2A. The white arrow indicates the direction of polarization before the objective lens. The yellow scale bar indicates 500 nm.

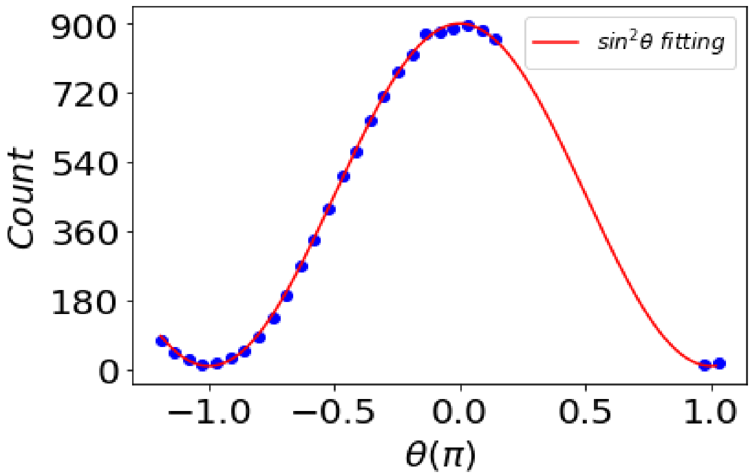

Supplementary Figure 8: Transmission intensity versus angle of the transmission axis of a linear polarizer. We placed a linear polarizer on the sample plane and recorded the transmission light while rotating the linear polarizer. The vertical axis indicates the signal recorded by transmission PMT and the horizontal axis indicates the angle of the optical axis of the polarizer with respect to the x axis of the sample plane. The blue dots are the measured data and the red line is the $sin^2\theta$ function fitting. The ratio between y-polarized and x-polarized light is about 1%, manifesting the linearly polarized light.

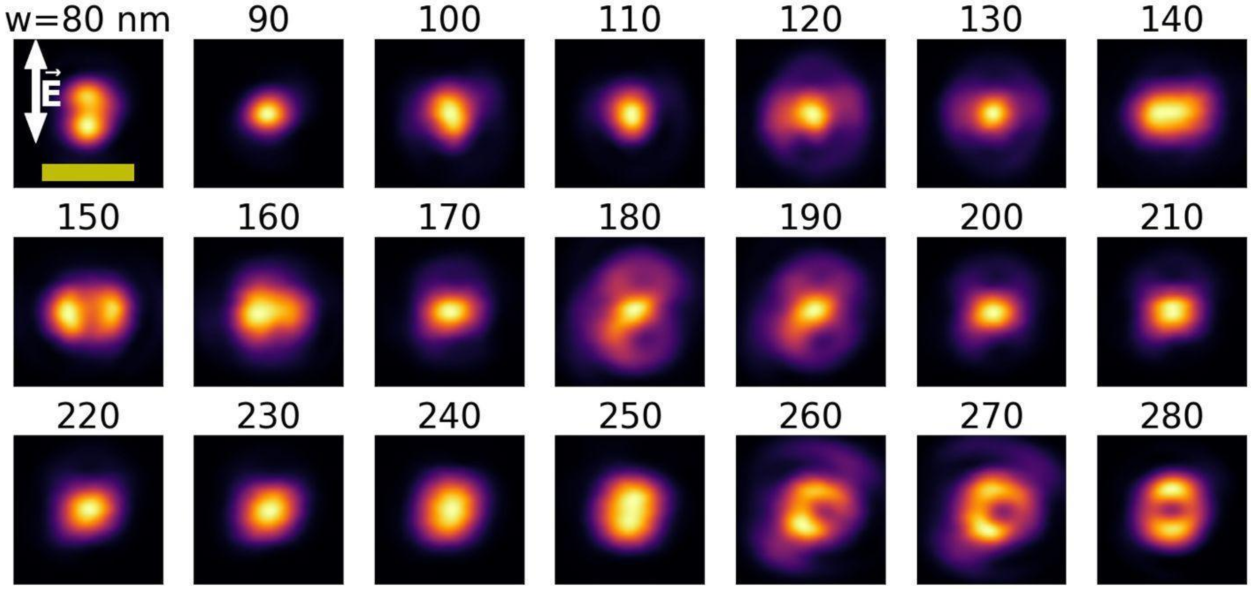

Supplementary Figure 9: Experimental LSM images of the whole array at vertical polarization, manifesting the image polarization dependency when compared with Fig. 2a. The white arrow indicates the direction of the electric field before the objective lens. The yellow scale bar indicates 500 nm.

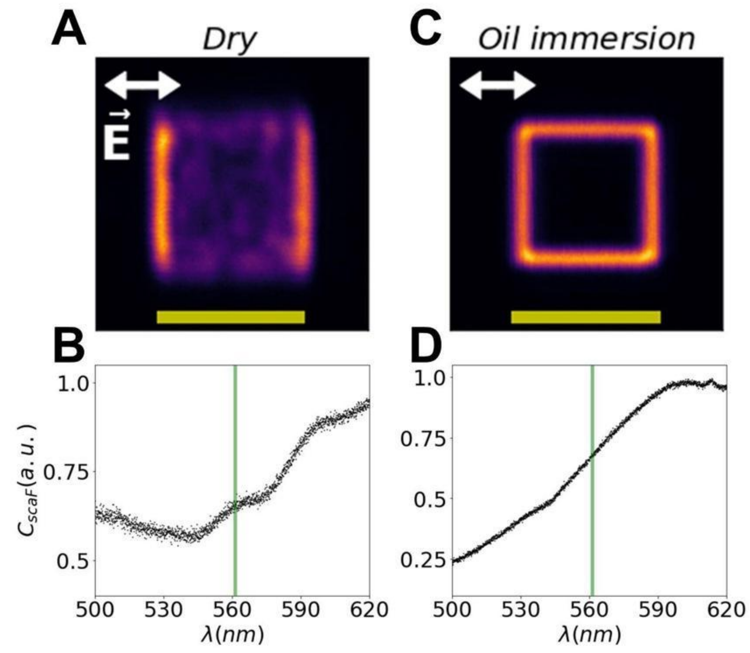

Supplementary Figure 10: LSM images and scattering spectra of a 10 µm wide Si thin film based on a low-NA objective, and with different immersion media. (A) Forward dark-field LSM image of Si thin film in air with loosely focused condition. The measured focal spot FWHM is 1.2 µm, i.e. w/FWHM ~ 8. The laser polarization is horizontal, and only the edge that is perpendicular to the polarization shows strong scattering, manifesting the idea of displacement resonance in the central panel of Fig. 1a. **(B)** Forward dark-field scattering spectrum at the edge of the dry Si thin film, showing a Mie resonance peak located at the 561 nm excitation wavelength. **(C)** Forward dark-field LSM image of the same Si thin film, but immersed in oil, with the same focusing condition. Interestingly, now all edges show scattering. **(D)** Forward dark-field scattering spectrum of the oil-immersed Si thin film. There is no Mie resonance peak at 561 nm. The yellow scales in **(A)** and **(C)** are 10 µm.

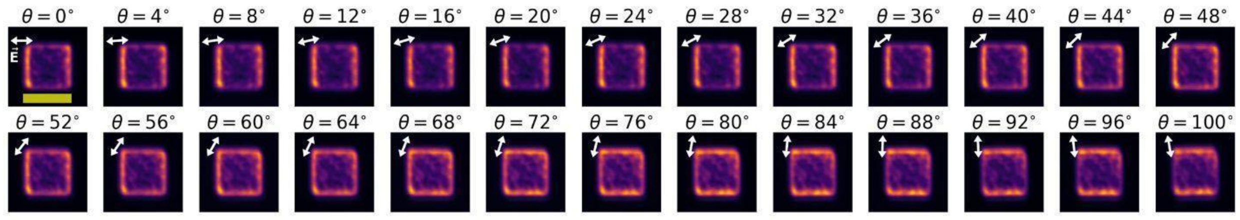

Supplementary Figure 11: Polarization-dependent forward scattering LSM images of the 10-µm wide Si thin film in air. Following the result in Fig. S7, here the title on each panel indicates the polarization orientation with respect to the x-axis, as illustrated by the white arrows. The yellow scale bar indicates 10 µm.

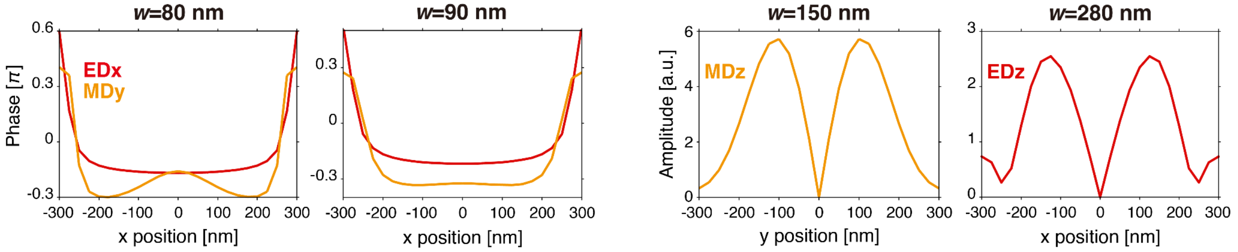

Supplementary Figure 12: Multipole decomposition analysis (MDA) of four representative particles with focus displacements. Left two panels are dependences of scattering phase on the x-position of focus spot for w = 80 nm and 90 nm silicon nanocuboids. Right two panels are z-components of MD and ED mode amplitudes for w = 150 nm and 280 nm silicon nanocuboids, respectively.

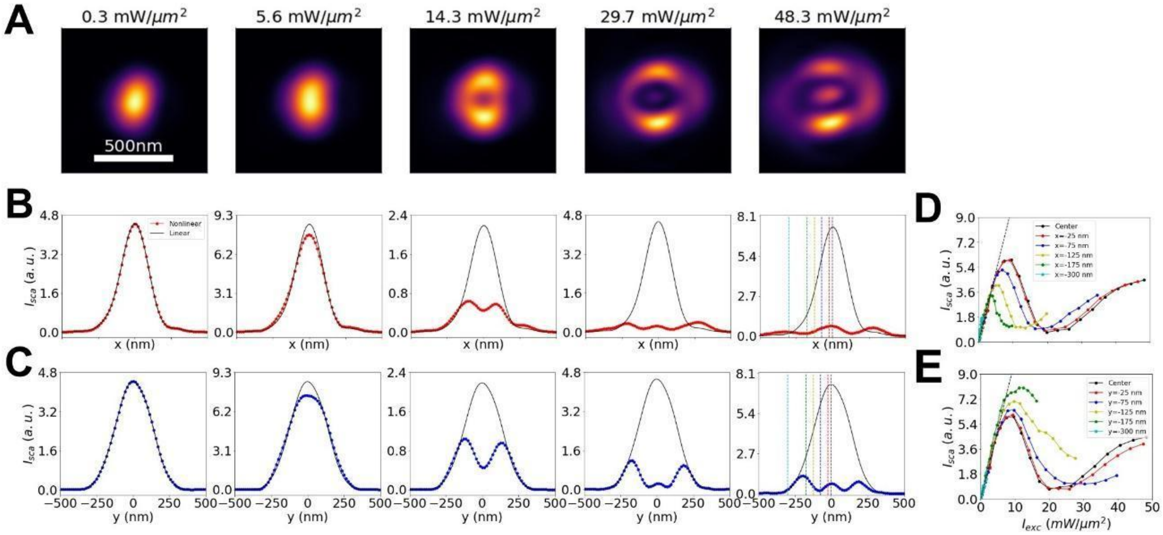

Supplementary Figure 13: Line profile photothermal scattering nonlinearity analysis. **(A)** shows laser scanning images with increasing excitation intensities, from 0.3 mW/μm^2 to nearly 50 mW/μm^2. **(B)** and **(C)** present the corresponding scattering intensity profiles along the x and y directions, respectively. At low intensity, only linear interaction is expected, and the image profile is well-fitted by a Gaussian function. As the excitation intensity increases to around 5 mW/μm^2, scattering slightly reduces from the center, indicating that the nonlinear response starts at the peak location. Significant reduction of scattering is observed when further increasing the excitation intensity. At 30 mW/μm^2 and above, a peak arises from the center, suggesting the nonlinear behavior of reverse saturation[5]. (D) and (E) summarize the nonlinear power dependencies at different focus displacements, i.e. 25, 75, 125, 175, and 300 nm away from the nanoparticle center, as marked by the five vertical lines in the last panel of (B) and (C), respectively. The x=300 nm curve, where the maximum excitation intensity is below 3 mW/μm^2, verifies the linear power dependency of the experimental system.

Supplementary References:

1. Agrawal, G. P. & Lax, M. Free-space wave propagation beyond the paraxial approximation. *Phys. Rev. A Gen. Phys.* **27**, 1693–1695 (1983).
2. Bohren, C. F. & Huffman, D. R. *Absorption and scattering of light by small particles*. (John Wiley & Sons, 1998).
3. Alaee, R., Rockstuhl, C. & Fernandez-Corbaton, I. An electromagnetic multipole expansion beyond the long-wavelength approximation. *Opt. Commun.* **407**, 17–21 (2018).
4. Duh, Y.-S. *et al.* Giant photothermal nonlinearity in a single silicon nanostructure. *Nat. Commun.* **11**, 4101 (2020).
5. Chu, S.-W. *et al.* Saturation and Reverse Saturation of Scattering in a Single Plasmonic Nanoparticle. *ACS Photonics* **1**, 32–37 (2014).
