# Supplementary material for: Multipole engineering by displacement resonance: a new degree of freedom of Mie resonance
Source: Nat Commun. 2023 Nov 8;14:7213. doi: 10.1038/s41467-023-43063-y (PMC10632421; doi:10.1038/s41467-023-43063-y)
Supplement: Supplementary file 4 — Description of Additional Supplementary Files [file 41467_2023_43063_MOESM4_ESM.pdf]

Description of Additional Supplementary Files:

Supplementary Movie 1: Size-dependent scattering images

Supplementary Movie 2: Power-dependent nonlinear LSM images
